# Influence of different final irrigation protocols for removal of photosensitizer activated by ultrasonic device on the bond strength of gutta-percha/bioceramic sealer and fiberglass posts/self-adhesive cement

**DOI:** 10.1007/s10103-026-04838-z

**Published:** 2026-03-02

**Authors:** Vitor Hugo Sanches Menchik, Anna Vithoria Da Costa Longhi, Matheus Albino Souza, Alexia Trento, Bianca Ávila Bratz, Maria Eduarda Klaesener, Marianna Demarchi, Karol Eduarda Bordignon Möhr, João Paulo De Carli, Yuri Dal Bello

**Affiliations:** Universidade de Passo Fundo, Passo Fundo, Brazil

**Keywords:** Bond strength, EDTA, Glycolic acid, Photodynamic therapy, Ultrasonic activation

## Abstract

The aim of present study was to evaluate the influence of final irrigation protocols for removal of photosensitizer *activated* by ultrasonic device(US) on the bond strength(BS) of gutta-percha/bioceramic sealer and fiberglass posts/self-adhesive cement to root dentin. One hundred single-rooted teeth were used, being 50 roots used to assess the BS of filling material and 50 to assess the BS of restorative material. After sample preparation, photodynamic therapy with US was performed, using 0.01% methylene blue as photosensitizer and diode laser as light source. The 50 roots of each evaluation were randomly divided into five groups (n=10), according to irrigation protocol for photosensitizer removal: G1 – distilled water + US; G2 – 17% EDTA; G3–17% Glycolic acid(GA); G4 – 17% EDTA + US; G5 – 17% GA+US. Then, 50 roots were filled with gutta-percha/Bio-C bioceramic sealer and 50 roots were filled with fiber glass posts/Rely-X U200 self-adhesive cement. In both, the roots were sectioned to obtain 1 mm thick dentin/material discs, and the push-out test was performed. Failure patterns were observed under optical microscope. Specific statistical analysis was performed (α=5%). In both evaluations, BS was significantly higher in groups 4(17% EDTA + US) and 5(17% GA + US) when compared to all other groups(*p* < 0.05). Regarding the failure patterns, no statistically significant difference was found between groups (*p* > 0.05), with a predominance of cohesive failure in all groups. The association of US with EDTA and GA for photosensitizer removal improved the BS of filling/restorative material to root dentin.

## Introduction

 Photodynamic therapy (PDT) represents an auxiliary disinfection resource in the endodontic field, involving the ability of a photosensitizer to absorb light energy and react with oxygen, generating reactive oxygen species. These species adhere to and penetrate the bacterial cell wall, precipitating cytoplasmic content and inducing damage to bacterial DNA [1]. It represents an alternative in the disinfection process, complementing conventional chemomechanical preparation and assisting in the reduction of bacterial species inside the root canal.

 Similarly, ultrasonic activation (US) has been explored as an adjunctive method to enhance disinfection in endodontic treatment, contributing to microbial reduction through increased temperature and hydrostatic pressure of the irrigant solutions used within root canals [2]. Considering the microbial etiology of pulp and periapical pathologies, the hydrodynamic turbulence induced by these physical mechanisms of US has also been recommended to assist conventional chemical-mechanical preparation, providing higher penetration and root canal disinfection [1, 2].

 According to Ghinzelli et al. [3], the use of US over the photosensitizer of PDT resulted in higher elimination of bacteria from the root canal space. However, this US can contribute to a significant reaching, penetration, and adhesion of the photosensitizer to the canal walls. It can act as a chemical smear layer, creating a strongly adhered barrier and making its removal from the root canal walls more difficult, taking into account the subsequent filling and restoration of the endodontically treated tooth. The photosensitizers present hydrophilic nature, low molecular weight, and high viscosity. It creates a chemical smear layer that strongly adheres to the canal walls and dentinal tubules [4], reducing the bond strength of filling and restorative materials [5, 6]. In addition, the US can induce greater impulse and impregnation of the photosensitizer to the canal walls, further compromising adhesion. Therefore, the use of final irrigation protocols should be considered when photodynamic therapy is performed.

 The ethylenediaminetetraacetic acid (EDTA) and glycolic acid (GA) are final irrigants with the ability to remove the smear layer [7, 8]. The US of these final irrigants contribute for higher smear layer removal [9]. In order to overcome certain drawbacks of EDTA, GA has been used for comparison as an alternative final irrigant. It includes higher cytotoxicity and reduction of mechanical properties from the use of EDTA as a final irrigant [7, 8]. Thus, the association of GA with US could represent an alternative for photosensitizer removal after PDT, in order to provide satisfactory adhesion of filling/restorative materials.

 Recent research has demonstrated scientific evidence for the use of bioceramic sealers as filling materials [10]. Among other properties, it demonstrates adequate bond strength to dentin, even after irradiation protocols, such as PDT [11]. At the same time, the use of self-adhesive resin cement has been recommended to promote better bond strength of glass fiber posts (GFPs) to the root dentin when the coronal portion presents extensive loss [12]. However, there are no studies in the literature revealing the effectiveness of final irrigation protocols for photosensitizer removal, when the photosensitizer was activated by US. Moreover, the influence of these protocols on the bond strength of bioceramic sealers and self-adhesive resin cements is not well elucidated.

The aim of the present study is to evaluate the influence of different final irrigation protocols for removal of photosensitizer activated by an ultrasonic device on the bond strength (BS) of gutta-percha/bioceramic sealer and glass fiber posts/self-adhesive cement to root dentin. The hypotheses of the present study were that US of the tested final irrigants provides effective removal of the photosensitizer, (i) improving the BS of filling and (ii) restorative materials to root dentin.

## Materials and methods

 The present study was designed according to the Checklist for Reporting In Vitro Studies (CRIS) guidelines [13]. This study was appreciated and approved by the local Ethics Commission under protocol 735.208.

### Sample collection and preparation

One hundred single-rooted extracted human teeth were used in the present study, with 50 teeth used to evaluate the BS of filling material, and 50 teeth used to evaluate the BS of restorative material. In each one of these evaluations, the 50 teeth were divided into five groups, with 10 teeth per group. From each group, five discs containing dentin/material were prepared, totaling 50 samples per group. The BioEstat 5.0 statistical package (Fundação Mamirauá, Belém, PA, Brazil) was used to calculate the sample size in all evaluation tests. A minimum of 30 samples per group was required to be included. The test power was considered to be 80% and the alpha error 5%, in order to estimate the sample size for analyzed outcomes.

All teeth were obtained from the Biobank of the School of Dentistry of the University of Passo Fundo (Passo Fundo, RS, Brazil). For this reason, the Human Ethics and Consent to Participate declarations are not applicable. Dental crowns were sectioned with a rotary diamond disc (#911H, Brasseler, Savannah, GA, United States) so that all roots retained a length of 15 mm. The root length of 15 mm was measured with a ruler, a mark was made on each root at the15 mm measurement and the cut was performed at this length, standardizing the length of all roots.

After this, 50 roots were prepared by a single operator, using the same protocol for pulp tissue removal and standardization of the root canal diameter. The working length (WL) was established by introducing a K-file #10 (Dentsply-Maillefer, Ballaigues, Switzerland) into the canal until its tip was visualized at the apical foramen. From this measurement, 1 mm was subtracted to obtain the WL. Each tooth was fixed in a portable lathe machine in order to maintain the tooth secured during the root canal preparation. The roots were enlarged to WL using the ProTaper system (Dentsply-Maillefer), following the sequence S1 to F3. Distilled water (DW) (Natupharma, Passo Fundo, RS, Brazil) was used as the irrigant solution and renewed at each instrument change. The ProTaper files (Dentsply-Maillefer) were used in a 16:1 gear reduction handpiece powered by a torque-controlled electric motor (X-Smart Plus - Dentsply-Maillefer, Ballaigues, Switzerland) at a constant rotation speed of 300 rpm in a crown-down manner according to the manufacturer’s instructions, using a gentle in-and-out digital motion.The remaining 50 roots were flared at their coronal and middle thirds using Gates Glidden drills no. 2, 3 and 4 to a depth of 10 mm, after chemo-mechanical preparation, in order to provide adequate space for cementation of glass fiber posts (GFPs). The Gates Glidden drills were used in a low speed handpiece powered by micro electric motor at a constant rotation speed of 10.000 rpm in a crown-down manner, by using a gentle in-and-out digital motion.

All root canals were then filled with 17% EDTA (Biodinâmica, Ibiporã, PR, Brazil), and all roots were placed into 10 mL plastic vials containing 17% EDTA, with ten samples per vial, so that the roots remained completely covered by the solution. Each plastic vial was inserted into an ultrasonic cleaning machine (Bio Free, Gnatus, Ribeirão Preto, SP, Brazil) for one minute. After that, the root canals were irrigated with 5mL of DW and dried with absorbent paper points (Tanari, Manacapuru, AM, Brazil). This protocol is recommended to remove the smear layer formed by root canal preparation, as well as to open the dentinal tubules and ensure satisfactory penetration of the photosensitizer in the PDT [4–6].

## PDT protocol with US

The 100 specimens were embedded in epoxy resin (Silaex, São Paulo, SP, Brazil) to facilitate the PDT protocol. The root canals were filled with 0.01% (0.1 mg/mL) methylene blue (Chimio Lux DMC, São Carlos, SP, Brazil) until extravasation at the root canal entrance. The photosensitizer was retained in the root canal for 5 min as pre-irradiation time. Then, US was performed using an ultrasonic device (Nac Plus Ultrasonics, Adiel, Ribeirão Preto, SP, Brazil). The stainless-steel E1 irrisonic endodontic tip (Helse Ultrasonic, Santa Rosa de Viterbo, SP, Brazil) was inserted 1 mm short of the WL and activated for 1 min. Scale power 1 for endodontics (25% power) was used to promote US. After that, a low-intensity laser (Therapy XT^®^ DMC, São Carlos, SP, Brazil) was used at 100 mW power and continuous emission in the red part of the spectrum (660–690 nm wavelength), with an intra-canal optical fiber of 600 μm diameter, attached 2 mm short of the working length. The root canals were irradiated for 90 s, with 9 J of total dose delivery and 320 J/cm2 of energy density, keeping the intra-canal fiber in a static position, as recommended by the manufacturer. Then, all roots were irrigated with 5 mL of DW, followed by aspiration of root canals.

## Classification of treatment protocols

According to experimental tests, 50 specimens were used for the evaluation of bond strength of filling material and the remaining 50 specimens were used for evaluation of bond strength of restorative material. In both evaluations, the 50 specimens were randomly divided into five groups (*n* = 10), according to the final irrigation protocol for photosensitizer removal: G1 – DW + US; G2–17% EDTA; G3–17% GA; G4–17% EDTA + US; G5–17% GA + US. The DW, 17% EDTA, and 17% GA were obtained from a compounding pharmacy (Natupharma, Passo Fundo, RS, Brazil).

In the groups without US, the root canals were completely filled with the tested solution until extravasation at the root canal entrance. Then, the tested solution remained in contact with root canal walls for a period of 1 min. After that, irrigation with 5 mL of DW was performed, thereby concluding the procedure for photosensitizer removal. In the groups with US, the root canals were completely filled with the tested solution until extravasation to the root canal entrance. Then, US was performed using an ultrasonic device (Nac Plus Ultrasonics, Adiel, Ribeirão Preto, SP, Brazil). The stainless-steel E1 irrisonic endodontic tip (Helse Ultrasonic, Santa Rosa de Viterbo, SP, Brazil) was inserted 1 mm short of the WL and activated for 1 min. Scale power 1 for endodontics (25% power) was used to promote US. Every effort was made to minimize contact of the tip with the root canal walls and to promote the agitation of the tested final irrigant. After this, irrigation with 5 mL of DW was performed, thereby concluding the procedure for photosensitizer removal.

All root canals were dried with an aspiration cannula and absorbent paper points (Tanari, Manacapuru, AM, Brazil) in both evaluations.

## Root canal filling

In the first evaluation (BS of the filling material), the 10 specimens of each of the five groups were filled by the lateral compaction technique using gutta-percha points (Dentsply-Maillefer) and Bio-C bioceramic sealer (Angelus, Londrina, PR, Brazil). The sealer was applied along the root canal walls using the insertion point of bioceramic sealer. The gutta-percha ProTaper master cone #F3 (Dentsply-Maillefer) was lightly coated with the bioceramic sealer and inserted to the WL. Then, XF gutta-percha accessory points (Dentsply-Maillefer) were introduced into the root canals with the aid of finger spreaders (Dentsply-Maillefer, Ballaigues, Switzerland). The gutta-percha accessory points were used until the finger spreader did not penetrate more than 5 millimeters into the root canal. Then, the excess of filling material was removed by cutting with a #2 heated plugger (SS White Duflex, Rio de Janeiro, RJ, Brazil). The root canal entrance was sealed with temporary restorative material (Vidrion R - SS White, Rio de Janeiro, RJ, Brazil).

In the second evaluation (BS of the restorative material), the 10 specimens of each of the five groups were filled with GFPs (Angelus, Londrina, PR, Brazil) and Rely-X U200 self-adhesive cement (3 M ESPE, St. Paul, MN, USA). The GFP no. 1 (Angelus) was cleaned with 35% phosphoric acid for 30 s, rinsed for 30 s, and gently air-dried. The silane application (3 M ESPE) was performed for 1 min, followed by Single-Bond adhesive application (3 M ESPE) and light polymerization for 40 s with a halogen light source with a power of 600 mW/cm^2^ (Optilux, Demetron Res. Corp, Danbury CT, USA). The Single-Bond adhesive (3 M ESPE) was applied to the root dentin walls using microbrushes, air-dried for 5 s, and light polymerized for 40 s. Subsequently, Rely-X U200 self-adhesive cement (3 M ESPE) was mixed and injected into the root canal of the 10 samples of each group with a suitable Centryx syringe and Acudosse needle (DFL, Rio de Janeiro, RJ, Brazil). The GFP was then covered with the same cement and positioned within the root canal at a 10 mm level, and held under digital pressure for 20 s. After this period, excess cement was removed. The cement was then polymerized using a 600 mW/cm^2^ halogen light source (Optilux) for 30 s on each face (buccal, palatal, mesial, distal, and occlusal).

All procedures and parameters from this section were based on the manufacturer’s instruction.

## Evaluation on bond strength

After the filling procedures, all specimens were stored at 37 °C and 95% humidity for 21 days. Subsequently, the roots were sectioned transversely from the root canal entrance into 1 mm thick discs using a metallographic cutter with a diamond disk, at a speed of 350 rpm under cooling. The first disc was discarded, and the next five root discs were selected from each sample, totaling 50 specimens per subgroup (*n* = 5 × 10 = 50). Each disc was subjected to the push-out test on a mechanical testing machine (Emic DL 2000, São José dos Pinhais, PR, Brazil) at a speed of 1 mm/min using a stainless steel cylindrical plunger of 0.8 mm diameter. The plunger tip was positioned so that it only contacted the filling material. The push-out force was applied in an apico-coronal direction until bond failure occurred, which was manifested by extrusion of the filling material and a sudden drop along the load deflection. The force required to displace the material from the root canal was recorded in Newtons (N) and calculated in megapascals (MPa).

After the push-out test, each disc was immediately transported in an Eppendorf tube containing DW to the optical microscope site, in order to calculate the BS and evaluate the failure patterns. Each disc was removed from the Eppendorf tube with a tweezer, dried with absorbent paper, and positioned in the center of the optical microscope (Zeiss, São Paulo, SP, Brazil), at 50× magnification. The BS calculation and the failure pattern evaluation were based on a previous study by Dias et al. [14]. The BS (δ) in megapascals was calculated using the formula δ = F/A, in which F is the force (N) applied by the test machine and A is the area. To calculate the area, the following equation was applied: A = 2πr × h, in which π is the constant value 3.14, r is the radius of the intra-radicular space, and h is the height (mm). The radius of the intra-radicular space of each disc was measured under optical microscopy, with the aid of software which provided this measurement. The height of each disc was measured using a digital pachymeter. Furthermore, the failure patterns were observed in each disc under optical microscopy (Zeiss, São Paulo, SP, Brazil) at 50× magnification. The classification was established as follows: 1: adhesive, between the dentin and the filling/restorative material, absence of filling/restorative material on the dentin walls of the root canal; 2: cohesive, failure of the filling/restorative material, presence of filling/restorative material on the dentin walls of the root canal; and 3: mixed, both failures (1 and 2) could be observed. All procedures and parameters from this section were based on the previous study of Dias et al. [14].

### Statistical analysis

The normal distribution of results was confirmed by the Kolmogorov–Smirnov test (*p* = 0.4015). The BS was evaluated using a one-way analysis of variance (ANOVA), followed by the Tukey post-hoc test, enabling a quantitative analysis of these data. The failure mode distribution among the groups was evaluated using the chi-squared test, enabling a descriptive analysis of these data. All tests were set at a 5% level of significance. The effect size was calculated according to Cohen’s criteria. Data were analyzed using Stat Plus AnalystSoft Inc., version 6.0 (Vancouver, BC, Canada).

## Results

The mean and standard deviation of BS of the filling and restorative material to the root canal dentin after the tested protocols are presented in Tables 1 and 2, respectively. According to results of present study, the BS of filling material (gutta-percha/Bio-C bioceramic sealer) and restorative material (GFPs/ Rely-X U200 self-adhesive cement) were exactly the same, with higher BS in groups 4 (17% EDTA + US) and 5 (17% GA + US), with statistically significant differences when compared to the other groups (*p* < 0.05), whereas there were no statistically significant differences between them (*p* > 0.05). In addition, the BS of filling and restorative materials were higher in groups 2 (17% EDTA) and 3 (17% GA), with statistically significant differences when compared to group 1 (DW + US) (*p* < 0.05), whereas there was no statistically significant differences between them (*p* > 0.05). The groups 4 (17% EDTA + US) and 5 (GA + US) presented higher bond strength values, with high effect size after calculation (Cohen’s d = 1.02). Finally, no statistically significant differences were revealed in failure patterns among the groups (*p* > 0.05), with a higher predominance of cohesive failure in all groups of both evaluations.

**Table 1:** Mean (standard deviation) of bond strength of filling material to root Canal dentin (MPa) and percentage of pattern of failure (%) after tested final irrigation protocols

Group	*n*	Push Out Bond Strength	Failure mode		
			Adhesive	Mixed	Cohesive
1. DW + US^a^	30	4.62 (2.51)	10.00	33.33	56.67
2. EDTA^b^	30	16.10 (4.42)	10.00	23.33	66.67
3. GA^b^	30	20.61 (5.13)	13.34	33.32	53.34
4. EDTA + US^c^	30	29.95 (3.23)	16.67	33.33	50.00
5. GA + US^c^	30	33.35 (3.96)	26.67	19.99	53.34

*Different superscript lowercase letters indicate, in the column, statistically significant differences (*p* < 0.05)**DW, distilled water; US, ultrasonic activation; EDTA, Ethylenediaminetetraacetic acid; GA, glycolic acid

**Table 2:** Mean (standard deviation) of bond strength of restorative material to root Canal dentin (MPa) and percentage of pattern of failure (%) of tested final irrigation protocols

Group	*n*	Push Out Bond Strength	Failure mode		
			Adhesive	Mixed	Cohesive
1. DW + US^a^	30	4.90 (1.83)	6.67	39.99	53.34
2. EDTA^b^	30	21.43 (3.99)	10.00	26.66	63.34
3. GA^b^	30	22.55 (4.41)	6.67	33.33	50.00
4. EDTA + US^c^	30	30.95 (3.74)	10.00	40.00	50.00
5. GA + US^c^	30	33.70 (3.02)	10.00	36.66	53.34

*Different superscript lowercase letters indicate, in the column, statistically significant differences (*p* < 0.05)**DW, distilled water; US, ultrasonic activation; EDTA, Ethylenediaminetetraacetic acid; GA, glycolic acid

## Discussion

The association of PDT and US represents an auxiliary resource to provide microbial reduction from the root canal system [3]. Although this benefit exists, there is a possibility that US may contribute to an even greater impregnation of the photosensitizer on the root dentin, which can reduce the BS of filling and restorative materials to root dentin. It is possible because the photosensitizer adheres strongly to the root canal walls and acts as a chemical smear layer [4]. Moreover, the association of US provides agitation of the photosensitizer, propelling this substance to the root canal walls and into the depth of the dentinal tubules, which can further increase this impregnation. Considering that the conventional PDT protocol forms a chemical smear layer that compromises the adhesion of filling and restorative material [5, 6], the present study was carried out to evaluate whether the US of the photosensitizer reduces the BS of the tested materials, even after the use of the tested final irrigation protocols.

The literature reveals that the association of 17% EDTA with US for 1 min results in effective removal of the photosensitizer from the root canal walls, as this irrigation protocol increases the bond strength of the filling/restorative materials to the root dentin [5, 6]. For these reasons, this irrigation protocol and contact time were used as a parameter in the present study. Furthermore, the present study proposes the use of 17% GA as a final irrigant, making a comparison with the 17% EDTA solution. According to Cecchin et al. [15], GA was tested as a surface pretreatment agent for dental restorative applications, presenting effective results on enamel etching and dentin surfaces. At the same time, GA revealed effectiveness for smear layer removal [8]. Thus, GA was chosen to be tested in the present study, with and without US, to evaluate its ability to promote the removal of the chemical smear layer formed by the US of the photosensitizer, and consequently increase the bond strength of the tested materials to root dentin.

The intracanal decontamination protocols performed prior to root canal filling or adhesive cementation of intraradicular posts must provide ideal conditions for BS to root dentin. This ensures effective adhesion to the root canal walls, minimizing marginal infiltration and the risk of root fracture, contributing to the longevity of endodontically treated teeth [16]. Considering that BS corresponds to the force required to displace the filling or restorative material adhered to the root dentin, the push-out test has been recommended over time to evaluate this mechanical property. It consists of applying a force to the filling/restorative material through a cross-section of the root until this material is displaced. The displacement force is uniform and simulates clinical reality, it can be performed in different thirds of the root canal, it has high reproducibility, and provides a larger tested adhesion area when compared to other tests, such as microtensile and shear tests [14, 17]. For these reasons, the push-out test was used in the present study to evaluate the bond strength of the tested materials.

The Bio-C bioceramic sealer is bioactive and releases calcium ions, providing sealing ability through stable chemical bonding, tag-like penetration into the depth of dentinal tubules, and stimulation of biomineralization [9, 18]. In turn, the Rely-X U200 self-adhesive resin cement provides recognized micromechanical and chemical retention to root dentin [19]. Considering that the US of the photosensitizer may impregnate root dentin and interfere with the adhesion of filling and restorative materials, the choice of endodontic sealer and adhesive cement for GFP plays a key role at this stage of endodontic treatment. Due to previously described properties, the Bio-C bioceramic sealer with gutta-percha and Rely-X U200 with GFP were tested in the present study after the root dentin was subjected to PDT associated with US, as well as after the tested final irrigation protocols for photosensitizer removal.

According to the results of present study, the bond strength was significantly higher in groups 2 (17% EDTA) and 3 (17% GA) when compared to control group. This is in agreement with the results of previous studies, where the use of final irrigants with ability to remove the smear layer after PDT protocol induced an increase in the bond strength of filling/restorative materials to root dentin [5, 6]. At the same time, the literature reveals that irrigation with inert solution is not enough for photosensitizer removal, leaving a chemical smear layer in the root canal walls [4]. It highlights the importance of introducing final irrigation techniques in the step-by-step of PDT protocol. Nowadays, it is not recommended. The EDTA acts by demineralizing of superficial dentin and decalcifying the root dentin [7]. The GA acts by acidic demineralization and indirect organic dissolution [8]. Therefore, both alternatives have the ability for effective photosensitizer removal from root canals, helping to improve the BS to root dentin.

The association of US with 17% EDTA and 17% GA resulted in the highest BS values for filling and restorative materials to root dentin, based on the results of present study. It confirms the first and second hypothesis of present study. The US acts through the principle of hydrodynamic turbulence, increasing the temperature and hydrostatic pressure of the irrigant inserted into the root canal. Bubbles and cavitations are then generated, and the irrigant agent is propelled more effectively against the root canal walls. It increases its cleaning potential and penetration into the depth of the dentinal tubules [20]. Similar results were observed in previous studies, also revealing that US promotes higher photosensitizer removal when compared with the isolated use of final irrigants with chelating properties, such as EDTA and QMix [5, 6]. According to van der Sluis et al. [2], the US represents a more effective supplement for cleaning the root canal system and root canal walls, when compared with traditional syringe irrigation. Therefore, the association of US with final irrigants must be considered an essential step into the PDT protocol, considering the findings of the literature and the results of present study.

Cohesive failure occurs within the filling or restorative material itself and not at the sealer-dentin or cement-dentin interface. After the push-out test in both evaluations, it was possible to observe a higher predominance of cohesive failure in all groups of present study. The Bio-C bioceramic sealer releases calcium and hydroxyl ions, which react with dentin phosphate and form hydroxyapatite at the sealer-dentin interface, creating micromechanical retention. This bioceramic sealer also exhibits volumetric expansion that fills microspaces and increases marginal adaptation [1, 11, 18, 21]. In turn, Rely-X U200 self-adhesive resin cement releases acidic monomers that demineralize and infiltrate the dentin substrate, providing micromechanical retention. At the same time, the reaction between the phosphoric acid monomers of the cement and hydroxyapatite in the dentin substrate provides chemical retention [19, 22]. All these mechanisms help to explain the high adhesion provided by the tested bioceramic endodontic sealer and self-adhesive resin cement, as well as the observed failure patterns in the present study.

Despite the fact that there is no statistically significant difference between EDTA and GA in the removal of the US-activated photosensitizer, as well as the fact that there is similarity in the potential for removing the smear layer formed by the instrumentation between the two final irrigants, GA presents some advantages over EDTA. The GA exhibits low cytotoxicity and does not induce severe damage to the mechanical properties of the dentin, even when activated by US, contrary to what is observed when EDTA is used, which can have deleterious effects in this regard [8, 23]. Considering this scenario, this study suggests the use of GA and US as part of the PDT protocol, ensuring effective photosensitizer removal from the root canal walls.

This in vitro study has some limitations. First, the experimental conditions do not fully reproduce the clinical environment, as factors such as biological fluids, functional stresses, and long-term aging were not simulated. Second, the use of standardized extracted teeth does not reflect the anatomical variability of root canal systems, which may influence both photosensitizer removal and BS outcomes. Finally, BS was evaluated only in the short term, without considering possible degradation of the dentin–sealer interface over time.

Further studies can be performed to evaluate the influence of different final irrigation protocols on photosensitizer removal and BS under simulated clinical conditions, including thermocycling, mechanical loading, and aging. The influence of these final irrigation protocols on the mechanical properties of root dentin also could be evaluated. In addition, investigations using root canals with greater anatomical complexity are needed to better reflect clinical scenarios. Finally, long-term studies assessing the durability of the dentin–sealer interface and the potential residual effects of photosensitizers over time are recommended. Based on new perspectives and tested variables, a safe protocol for removing the photosensitizer activated by US may be established.

## Conclusions

**Fig. 1:**
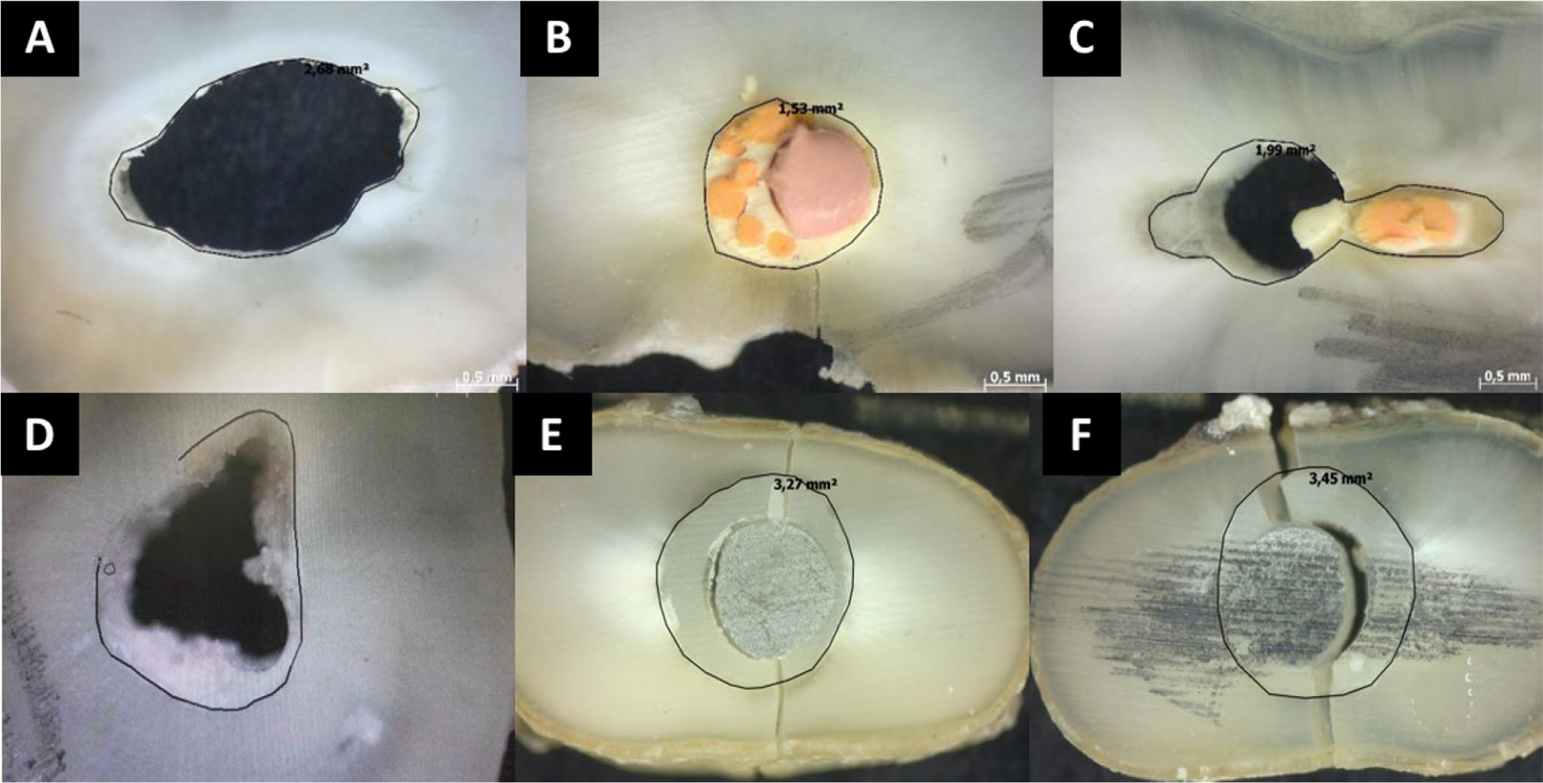
Illustrative images of failure patterns – **A**: adhesive, absence of filling material on the root canal walls; **B**: cohesive, presence of filling material on the root canal walls; **C**: mixed, both failures (adhesive and cohesive) observed in the disc containing filling material; **D**: adhesive, absence of restorative material on the root canal walls; **E**: cohesive, presence of restorative material on the root canal walls; **F**: mixed, both failures (adhesive and cohesive) observed in the disc containing restorative material

Despite the limitations of the present study, it can be concluded that final irrigation protocols using 17% EDTA + US and 17% GA + US effectively removed the photosensitizer activated by US, increasing the BS of gutta-percha/bioceramic sealer and glass fiber posts/self-adhesive cement to root dentin (Fig. 1).

## Data Availability

No datasets were generated or analysed during the current study.
